# Chemotherapy administration to breast cancer patients affects extracellular vesicles thrombogenicity and function

**DOI:** 10.18632/oncotarget.18792

**Published:** 2017-06-28

**Authors:** Anat Aharon, Anni Sabbah, Shahar Ben-Shaul, Hila Berkovich, David Loven, Benjamin Brenner, Gil Bar-Sela

**Affiliations:** ^1^ Department of Hematology and Bone Marrow Transplantation, Rambam Health Care Campus, Haifa, Israel; ^2^ Bruce Rappaport Faculty of Medicine, Technion-Israel Institute of Technology, Haifa, Israel; ^3^ Department of Oncology, Rambam Health Care Campus, Haifa, Israel; ^4^ Department of Oncology, Ha'emek Medical Center, Afula, Israel

**Keywords:** breast cancer (BC), extracellular vesicles (EVs), chemotherapy, thrombogenicity, endothelial cells (EC)

## Abstract

Breast cancer (BC) is the most prevalent type of malignancy in women. Extracellular vesicles (EVs) are subcellular membrane blebs that include exosomes and microparticles.

**Study aims:**

To elucidate the effects of chemotherapy administration on BC patients’ EVs characteristics and their effects on endothelial cells (EC) functions.

**Methods:**

EVs were isolated from the blood samples of 54 BC patients treated by chemotherapy (25 neo-adjuvant, 29 adjuvant) and from 20 healthy women (control group). Blood samples were taken before chemotherapy and on the day of last chemotherapy administration. In some patients, samples were also evaluated 24 hours after chemotherapy treatment. EVs were characterized by cell origin, thrombogenicity and cytokine content. EVs effects on coagulation, migration, apoptosis and proliferation of endothelial cells were assessed as well.

**Results:**

Patient characteristics of the two subgroups were similar except for tumor size. Change in EV expression of BC markers, MUC1 and EpCAM, were found in patient subgroups. EC-EVs were significantly higher in both patient subgroups compared to healthy controls. Higher EVs pro-coagulant activity was found at the end of chemotherapy and a significant increase in the ratio between tissue factor (TF) and TF pathway inhibitor was documented after the first 24hours of exposure to doxorubicin treatment. Furthermore, EVs of neo-adjuvant patients obtained before chemotherapy contained more pro-angiogenic proteins, reduced endothelial cells apoptosis and increased their migration compared to EVs obtained at the same timing from adjuvant patients.

**Conclusions:**

EVs may serve as a biomarker for chemotherapy-related thrombogenicity and may indicate vascular damage even before chemotherapy.

## INTRODUCTION

Breast cancer (BC) is the most prevalent type of malignancy among women [1, 2], and the risk for developing venous thromboembolism (VTE) is higher in individuals with BC than in the general population [3], resulting in a significant increase in morbidity and mortality [4]. That risk is increased along the chemotherapy period, mostly in the first month following its cessation, and in patients with metastatic disease [5].

Extracellular vesicles (EVs) are comprised of exosomes, intracellular luminal vesicles (50–90 nm), and microparticles (MPs) membrane vesicles (∼1mm in diameter) that shed from the cell surface of both normal and malignant cells, loading different types of cargo [6, 7]. EVs play a major role in intercellular communication within the tumor microenvironment and serve as a “vehicle” that modulates target cells by transferring proteins and genetic molecules (DNA, RNA, microRNA) [8]. These affect cell functions that are correlated in cancer patients with their tumor process, including differentiation, proliferation, migration, invasion and apoptosis, via changes in cell signaling pathways [9]. EVs also bear tissue factor (TF), the main activator of the coagulation cascade [10], and several anti-coagulant proteins, such as TF pathway inhibitor (TFPI) and endothelial protein C receptor (EPCR) [11]. Previous studies demonstrated that the number of EVs is higher in the plasma of BC patients in all stages and with various tumors sizes (T2-T4 tumors), in comparison to control groups [12], and were more thrombogenic [13].

In view of these observations, there is a need to elucidate the functional characteristics of EVs in cancer patients, and to clarify the potential effects of chemotherapy treatment on these characteristics. In the current study, we chose BC as a human model for the exploration of these needs. Patients who are diagnosed without demonstrable metastases are considered to bear micro-metastases. Therefore, high-risk patients at diagnosis receive chemotherapy aimed at reducing the risk of tumor recurrence and improving survival. This treatment is given either before surgical resection, as neo-adjuvant chemotherapy [14], or following surgical resection, as adjuvant chemotherapy [15]. The study aims were to explore the effects of primary tumor and various types of chemotherapy on the circulating vesicles quantities, their cell origins, cargo compositions, and their function in BC patients before and under chemotherapy, and to elucidate the effects of those EVs on endothelial cells thrombogenicity and functions. This was achieved both in patients with primary tumors (neo-adjuvant chemotherapy) and in patients who underwent surgical removal of their primary tumor (adjuvant chemotherapy). Thus, we theoretically were able to analyze the impact of the primary tumor and the impact of various types of chemotherapy on the circulating EVs.

## RESULTS

### Study population

Between October 2009 and November 2015, blood samples were collected from 54 BC patients treated by chemotherapy. The preoperative group (n=25) received neo-adjuvant treatment (median age 50 years, range 27-77 years) and the postoperative group (n=29) received adjuvant treatment (median age 57 years, range 35-81 years). In addition, blood samples were collected from 20 healthy women as a control group (median age 51 years, range 32-72 years). Patient characteristics are summarized in Table 1. Most patients (82%) were above age 40, and 80% had stage II or III disease. Only one preoperative patient versus 10 postoperative patients had stage I disease (*p*=0.016). Fifteen patients (nine postoperative, six preoperative) had no expression of hormone receptors and no human epidermal growth factor receptor 2 (HER2) overexpression (triple negative). All hormone receptor positive patients, except for one with early metastatic recurrence, received adjuvant hormonal therapy followed by radiotherapy treatment. All patients with HER2 overexpression (n=15) had been treated also with trastuzumab. Only 11 patients were tested for BRCA gene mutation, and six of seven in the postoperative group were found to be positive. In the preoperative subgroup, three patients had pathological complete remission confirmed by surgery, while three patients had clinical progression before surgery. All other preoperative patients had clinical partial response or stable disease. In a median follow-up of four years, three patients in the preoperative and one in the postoperative groups had systemic metastatic disease. Two in the preoperative subgroup passed away after 20 and 22 months. All other patients are without evidence of active disease. Two patients in the preoperative subgroup (8%, 2/25) had axillary or femoral deep vein thrombosis (DVT) during chemotherapy treatment. None of the patients with a medical history of VTE developed DVT during chemotherapy. The main side effects during chemotherapy were neutropenic fever in the Adriamycin and cyclophosphamide period (15%, 8/54 patients) and grade I-II peripheral neuropathy (according to Common Toxicity Criteria Adverse Events, version 3) during paclitaxel or docetaxel treatment (22%, 13/54 patients). There was no statistically significant difference between the two subgroups in any clinical or treatment parameter except for tumor size and stage of disease, higher in the preoperative subgroup (Table 1).

**Table 1:** Patient characteristics

		Preoperative	Postoperative
**Age**	<40 years	6	4
	>40 years	19	25
Medical history of VTE*		1	2
**Stage**	IA or IB	1, p=0.016	10
	IIA	9	9
	IIB	5	4
	IIIA	5	2
	IIIB or IIIC	5	4
**Grade**	I	1	0
	II	9	10
	III	12	18
	Unknown	3	1
**Tumor size**	<2 cm	0, p=0.0002	12
	2-5cm	12	15
	>5 cm	13, p=0.0001	2
**Number of lymph nodes involved**	0	7	12
	1-3	11	13
	4-9	5	3
	10+	2	1
**Estrogen receptor (ER)**	Negative	10	13
	Positive	15	16
**Progesterone receptor (PR)**	Negative	11	11
	Positive	14	18
**Triple negative breast cancer**		6	9
**Adriamycin and cyclophosphamide**	Given every 3 weeks	11	7
	Given every 2 weeks (dose dense)	14	18
	Not given	0	4
**Taxane base chemotherapy**	Not given	1	7
	Paclitaxel weekly for 12 weeks	18	14
	Paclitaxel every 2 weeks (dose dense)	2	8
	Paclitaxel weekly with carboplatin	4	0
	Docetaxel-carboplatin-Trastuzumab	0	4
**Operation**	Lumpectomy+SLNB**	3	8
	Lumpectomy+ALND***	13	9
	Mastectomy+SLNB	0	4
	Mastectomy+ALND	7	8
**Trastuzumab**	No	17	22
	Yes	8	7

Notes: * Venous-thrombus event.

** Sentinel lymph node biopsy.

*** Axillary lymph node dissection.

### EVs characterization

**Figure 1:**
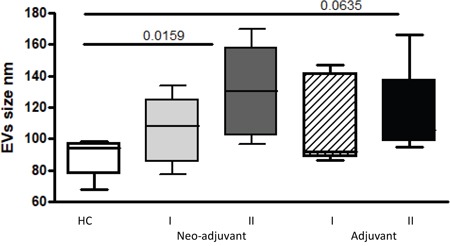
EVs size The size of circulating EVs (PPP) obtained from the study population were measured by Nanoparticle Tracking Analysis.

The average size of EVs was smaller in the healthy control group (88.9±12.3 nm) compared to patient EVs. A trend of increase in EVs size was found in EVs obtained from neo-adjuvant and adjuvant patients before chemotherapy (105.7±21.6 nm and 110.6±27.7 nm, respectively). Following chemotherapy, the size of EVs further increased, reaching statistical significance in the neo-adjuvant subgroup (130.1±29.5, *p*=0.0159), and close to significance in the adjuvant group (118±32.7, *p*=0.0635), as measured by NTA. However, EVs concentration remained similar in all study groups (Figure 1).

### EVs cell origin

### Tumor EVs

**Figure 2:**
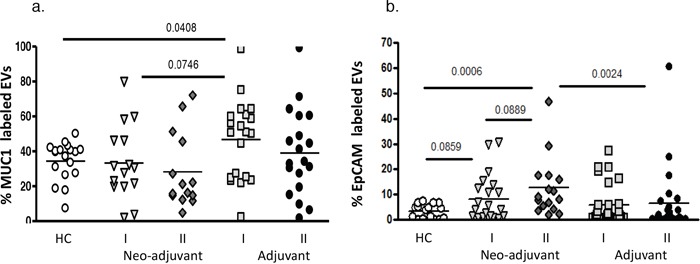
EVs tumorigenic markers EVs were isolated by a series of centrifugations. Antigen levels of tumorigenic markers MUC1 and EpCAM were measured on EVs obtained from healthy controls and on EVs obtained from patients before chemotherapy (time point I) and at the last chemotherapy treatment (time point II). The percentage of labelled EVs was calculated from the total number of EVs using FACS analysis. **(a)** MUC1, **(b)** EpCAM.

Mucin 1 (MUC1) levels in the healthy control EVs were similar to the neo-adjuvant EVs before chemotherapy (34.42±11.06% and 33.41±21.26%, respectively). Nevertheless, MUC1 levels were found to be significantly higher in the adjuvant group EVs (46.7±22.76%, *p*=0.041) before chemotherapy, and 52% of these adjuvant patients presented higher levels of EVs-MUC1 expression than the highest value found in controls. At the end of the chemotherapy, MUC1 levels were non-significantly higher in EVs obtained from adjuvant than in neo-adjuvant patients (39±24.7% and 28.2± 21.4%, respectively; *p*=0.074). However, the levels of EpCAM labeled EVs obtained from neo-adjuvant patients before chemotherapy demonstrated a trend of increase compared to healthy controls (8.2±9.2%, and 3.3±2.8%, *p*=0.086, respectively), without significant change compared to the EVs obtained from adjuvant patients before chemotherapy (5.7±7.9%). At the end of chemotherapy treatments, a trend of increase was documented in the EpCAM-EVs levels in the neo-adjuvant group (12.5±11.6%) compared to levels before chemotherapy (*p*=0.089) and significant higher levels compared to the adjuvant groups (6.7±13.3%, *p*=0.0024) (Figure 2).

### Endothelial EVs

**Figure 3:**
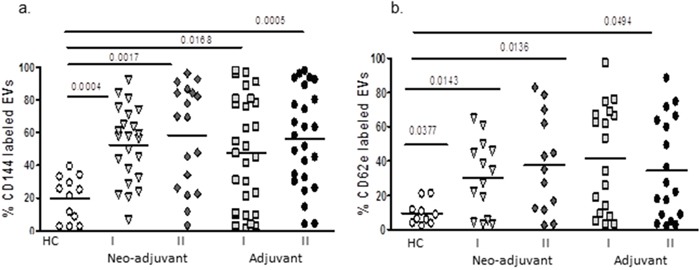
EVs endothelial markers EVs were isolated by a series of centrifugations. Antigen levels of endothelial markers VE-cadherin (CD144) and E-selectin (CD62E) were measured using specific fluorescent antibodies on EVs obtained from healthy controls and on EVs obtained from patients before chemotherapy (time point I) and at the last chemotherapy treatment (time point II). The percentage of labelled EVs was calculated from the total number EVs using FACS analysis.

Levels of CD144 (VE-cadherin), a marker for endothelial gap junction, were significantly lower on EVs obtained from the healthy controls group (19.6±13.5%) than EVs obtained from both patient groups before chemotherapy (neo-adjuvant 52.5±22.7%, *p*=0.0004 and adjuvant 47.7±33.7, *p*<0.0168, respectively). It was found that 69% of neo-adjuvant and 58% of adjuvant patients presented higher levels of EVs-CD144 expression than the highest value found in controls. No significant differences were found between patient subgroups before or at the end of the chemotherapy period, but 55% of neo-adjuvant and 52% of adjuvant patients presented an additional increase to their high levels of EVs-CD144 at the end of chemotherapy. The levels of EVs expressing CD62E, a marker for endothelial activation, obtained before chemotherapy from the neo-adjuvant and adjuvant groups were higher compared to EVs obtained from healthy control (30.2±21.5%, *p*=0.038; 41.5±31.1%, *p*=0.0136, 9.5±6.8%, respectively), whereas 57% of neo-adjuvant and 61% of adjuvant patients presented higher levels of EVs-CD62e than the highest value found in controls. This expression remained similarly high at the end of chemotherapy in both groups (37.8±28.5% and 34.7±29.3%, respectively), whereas 38% of neo-adjuvant and 27% adjuvant patients presented additional significant increase at the end of chemotherapy (Figure 3).

### EVs thrombogenicity

**Figure 4:**
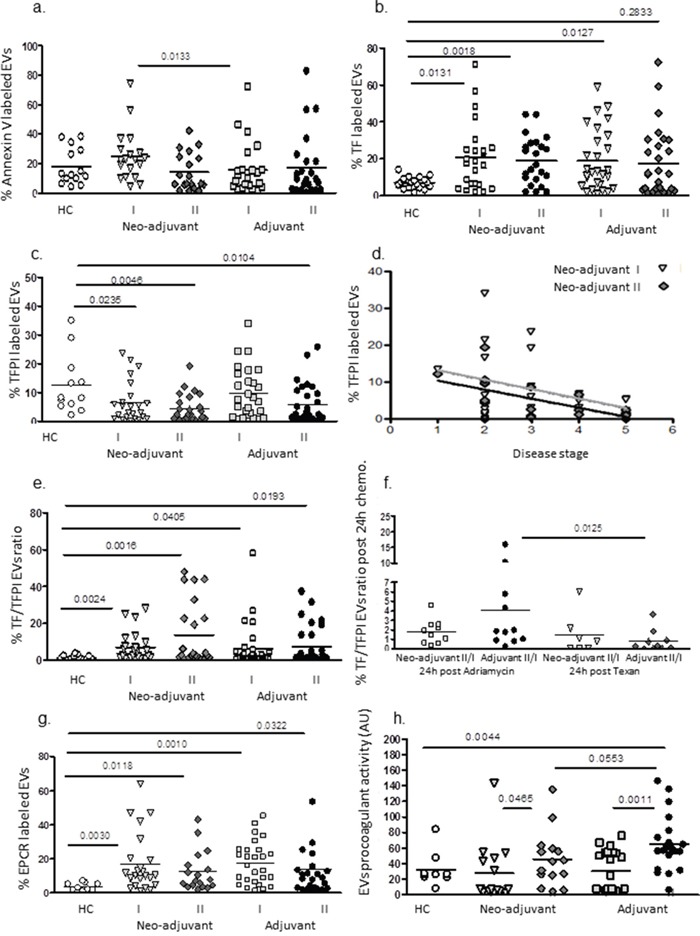
EVs thrombogenicity Levels of negatively charged phospholipids (labelled by Annexin V-FITC) and antigen levels of coagulation markers TF and TFPI were measured on EVs obtained from healthy controls and on EVs obtained from patients before chemotherapy (time point I) and at the last chemotherapy treatment (time point II), using specific fluorescent antibodies. The percentage of labelled EVs was calculated from the total number of EVs using FACS analysis and the ratio between TF and its inhibitor TFPI were calculated **(a-d)**. Correlation between EVs –TFPI expression and disease severity in neo-adjuvant patients at the end of chemotherapy was performed **(e)**. In addition, the change in TF/TFPI ratio after 24hours of first treatment with Adriamycin and cyclophosphamide or paclitaxel were measured as well **(f)**. Levels of EPCR on EVs **(g)**. Pro-coagulant activity of EVs was measured by the FXa chromogenic assay. Results are expressed as TF arbitrary units **(h)**.

The expression of negatively charged phospholipids on EVs membrane surface were measured by the percentage of Annexin V binding to EVs. The percentages before chemotherapy obtained in the neo-adjuvant group were significantly higher than in the adjuvant group (25.0±17.2 and 15.6±17.6, *p*=0.0133, respectively), but no significant differences were found between the subgroups at the end of chemotherapy or between patients and healthy controls (HC) (Figure 4a). The levels of the pro-coagulant protein, TF, were found to be significantly higher before chemotherapy in both groups (20.7±18.3, *p*=0.0131 and 19.0±16.5%, *p*=0.0127, respectively) compared to HC (7.0±3.1%). Higher TF levels than the highest values that were found in the controls were demonstrated in 55% of neo-adjuvant and 60% of adjuvant patients. During the chemotherapy period, the levels of TF did not change much and remained high in both patient subgroups; however, 48% of the neo-adjuvant and 30% of the adjuvant patients presented an additional significant increase in EVs-TF at the end of chemotherapy (Figure 4b).

In contrast, significant decreases were found in the expression of the inhibitor TFPI on EVs obtained from neo-adjuvant patients before chemotherapy and on EVs obtained from both patient subgroups (neo-adjuvant and adjuvant) at the end of chemotherapy (7.6±8.8%, *p*=0.023; 4.5±4.8%, *p*=0.0046 and 5.9±6.6%, *p*=0.0104, respectively) compared to EVs obtained from the HC group (12.5±10.3) without significant difference between subgroups (Figure 4c). In 58% of the neo-adjuvant patients and 55% of the adjuvant patients, the levels of TFPI bearing EVs additionally decreased at the end of chemotherapy. Moreover a significant inverse correlation was found between EVs –TFPI expression and disease severity in neo-adjuvant patients at the end of chemotherapy (Person r-0.60, 95% confidence interval −0.829 to −0.202, p=0.0065) (Figure 4d).

The ratio between TF and TFPI before chemotherapy on EVs obtained from neo-adjuvant patients (6.97±7.9 TF/TFPI ratio, *p*=0.0024) and on EVs obtained from adjuvant patients (6.38±11.8 TF/TFPI ratio *p*=0.0405) were higher compared to EVs obtained from HC (1.34±1.15 TF/TFPI ratio) without significant difference between patient subgroups (Figure 4e). Higher TF/TFPI ratio than the highest values that were found in the controls were demonstrated in 56% of neo-adjuvant and 31% of adjuvant patients before chemotherapy. In order to study the direct effects of chemotherapy on EVs thrombogenicity, TF/TFPI ratio was measured in some of the patients groups also after 24hours from the first Adriamycin/cyclophosphamide or paclitaxel treatment. We found a significant increase (>1.5-fold) in 6/10 patients in the neo-adjuvant group and in 7/10 patients in the adjuvant group after 24hours of doxorubicin treatment. In contrast, only 2 patient in each group demonstrated a significant increase in EVs-TF/TFPI ratio after treatment with paclitaxel (Figure 4f).

In addition, EPCR, a member of the protein C anticoagulant pathway that can act as a pro-thrombotic factor in its soluble form [16], was found in higher levels on EVs obtained before chemotherapy from neo-adjuvant and adjuvant patients (16.9±16.9%, *p*=0.003, and 17.4±12.5%, *p*=0.001, respectively) compared to EVs obtained from HC (3.7±2.2%) without differences between patient subgroups. At the end of chemotherapy, EPCR levels on the EVs remained similarly high in both subgroups (12.5±11.8% and 14.2±18.7%, respectively) (Figure 4g).

Levels of platelet EVs before chemotherapy were found to be similar in neo-adjuvant patients and HC (26.9±17.5% and 30.3±24.8%, respectively) and lower in the adjuvant group (16.5±16.1, *p*=0.045). At the end of chemotherapy, the neo-adjuvant and adjuvant platelet EVs were low without difference between the groups (18.5±19.9 and 17.5±18.2%, respectively). Levels of activated platelet EVs were low and similar across all study groups and time samplings.

Overall, patients’ EVs procoagulant activity before chemotherapy in both subgroups was found to be similar to EVs of HC, but chemotherapy induced a significant increase in the pro-coagulant activity of EVs obtained from both patient subgroups compared to EVs obtained before chemotherapy. EVs of the neo-adjuvant patients demonstrated a 1.5-fold increase, *p*=0.045, and EVs of adjuvant patients demonstrated a 2-fold increase, *p*=0.0011, as measured by activation of FX (Figure 4h).

### EVs growth factors and cytokines

**Figure 5:**
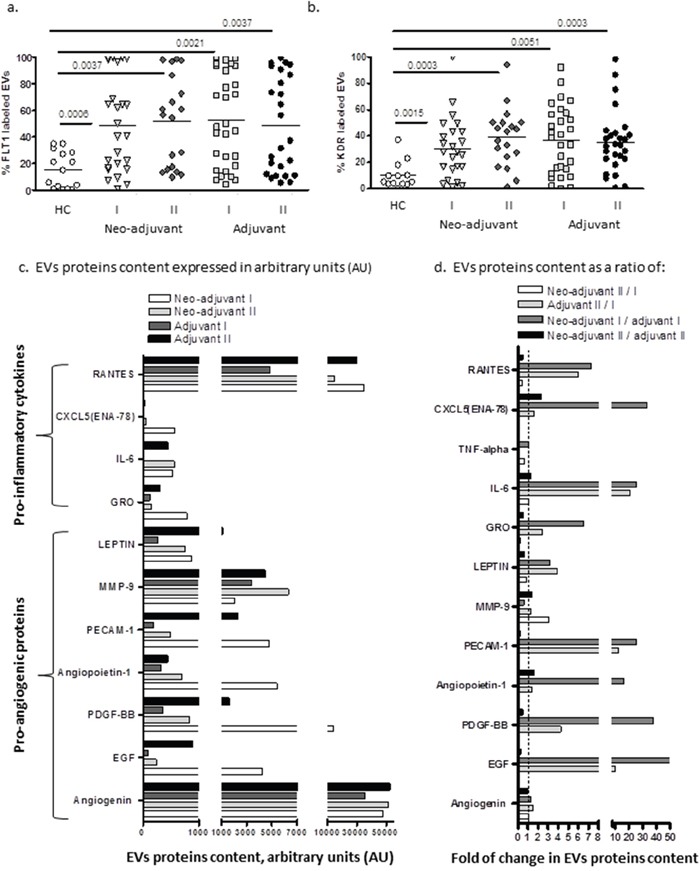
EVs expression of growth factors receptors and cytokines content Levels of growth factors receptors VEGFR1 (FLT1) and VEGFR-2 (KDR) were measured on EVs obtained from healthy controls and on EVs obtained from patients before chemotherapy (time point I) and at the last chemotherapy treatment (time point II), using specific fluorescent antibodies **(a, b)**. EV proteins extract was obtained from a pool of four specimens within each patient subgroup and validated by Human Angiogenesis Protein Antibody Array. Slides were analyzed using TotalLab software results. Each protein has significant signal intensities representing protein content, expressed in arbitrary units (AU) and presented in graph **(5c)**. The change in protein content in EVs obtained from the two subgroups at the same time point were calculated as a ratio between neo-adjuvant I/adjuvant I (dark gray bar) and neo-adjuvant II/adjuvant II (black bar). The effect of the chemotherapy on the EVs protein cargo was calculated in both subgroups as a ratio of neo-adjuvant II/neo-adjuvant I (white bar) and adjuvant II/adjuvant I (light gray bar) as presented in graph **(5d)**.

The levels of VEGF receptors 1 (FLT1) and VEGF receptors 2 (KDR) on the surface of EVs obtained from HC were low (FLT1-15±13.7% and KDR-10.2±10.3%) and found to be more than 3-fold higher in both patient groups before chemotherapy (neo-adjuvant FLT1: 48.5±35.3%, *p*<0.0006, KDR: 30.1±2.8, *p*<0.0015; adjuvant FLT1: 51.8±33.8%, *p*=0.0021, KDR: 36.6±25.6%, *p*=0.0051) and remained similarly high in both groups following chemotherapy (Figure 5a, 5b).

Screening of growth factors and cytokines contents in EVs demonstrated high variability between patient subgroups. Angiogenin, RANTES (Regulated on Activation, Normal T Cell Expressed and Secreted) and Matrix metallopeptidase 9 (MMP-9) were found to be the most abundant proteins in all patient EVs. While angiogenin levels remained unchanged in the end of chemotherapy, MMP-9 levels were increased in both patient subgroups, and RANTES level further increased only in the EVs obtained from the adjuvant patients (Figure 5c). In order to emphasize the differences between the protein cargo in EVs obtained from the two subgroups at the same time point, we calculated the ratio between the protein levels in EVs from neo-adjuvant patient/adjuvant patients before chemotherapy or at the end of chemotherapy. In order to emphasize the effect of the chemotherapy on the EVs protein cargo, we calculated the EVs protein ratio at the end of chemotherapy/before chemotherapy in each of the subgroups (neo-adjuvant or adjuvant) and differences between the protein cargo in EVs (Figure 5d). Protein screening demonstrated a significantly higher amount of several cytokines in EVs obtained before chemotherapy from neo-adjuvant patients than protein levels in EVs of adjuvant patients. This includes the growth factors, epidermal growth factor (EGF), platelet drive growth factor (PDGF), Angiopoietin-1, Platelet endothelial cell adhesion molecule (PECAM-1), leptin, and the growth related oncogene (GRO), and pro-inflammatory cytokines, such as interleukin 6 (IL6), CXC5, the Epithelial Neutrophil Activating Peptide-78, and RANTES (Figure 4d–dark gray bar). However, these high levels of cytokines in neo-adjuvant patient EVs were decreased to minimal levels at the end of chemotherapy (Figure 4d–white bar). EVs obtained from adjuvant patients at the end of chemotherapy demonstrated an increase in pro-inflammatory cytokines (Rantes and IL6) and in the growth factors EGF, PDGF, PECAM-1, compared to their levels before chemotherapy (Figure 4d–light gray bar) and compared to the content of EVs obtained from neo-adjuvant patients at the same time point (Figure 5d–black bar).

### EVs effects on endothelial cells (EC)

**Figure 6:**
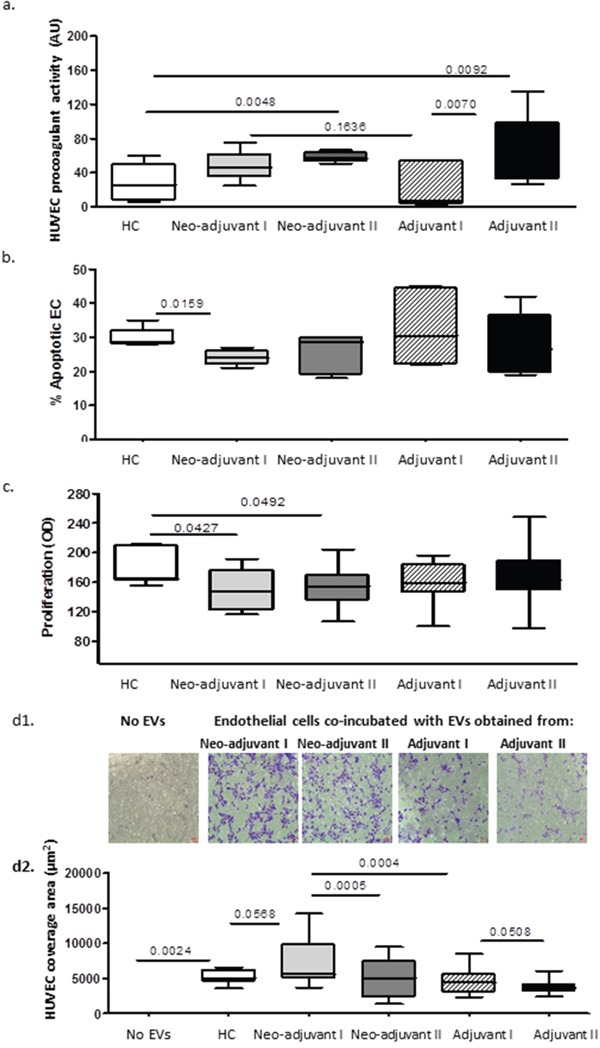
EVs effects on endothelial cells (EC) Human umbilical vein endothelial cells (HUVEC) were seeded for 20 hours with or without EV pellets (25μg) obtained from healthy controls (HC) and BC patients. **(a)** EV effect on EC thrombogenicity measured by FXa chromogenic assay. Results are expressed as TF arbitrary units. **(b)** EV effects on EC apoptosis measured by TUNEL assay. **(c)** EV effects on EC proliferation measured by the XTT assay. **(d)** EV effects on EC migration validated using the Boyden chamber. The area occupied by migratory cells was photographed by light microscopy (x10 magnification) **(d1)** and calculated using image J software **(d2)**.

Exposure of endothelial cells (EC) to patient EVs obtained after chemotherapy from both patient sub-groups significantly increased cell thrombogenicity. The effect of the adjuvant EVs was much more dominant compared to the neo-adjuvant EVs (Figure 6a). EC proliferation (Figure 6b) was decreased after exposure to patients EVs. This reduction was more significant after exposure to EVs from patients in the neo-adjuvant subgroup at time point I (p=0.0427) and at time point II (P=0.049) as compared to EC exposed to HC-EVs. In addition, apoptosis of ECs (Figure 6c) was reduced after exposure to EVs obtained from patients in the neo-adjuvant group at time point I as compared to EC exposed to HC-EVs (P=0.0159). On the other hand, migration of ECs was significantly increased at time point I, both after exposure to EVs from controls (by 35%, *p*=0.0568), and following exposure to EVs of patients in the adjuvant subgroup (58% increase, *p*=0.0004). EVs obtained at the end of the chemotherapy period (time point II) reduced the migration of EC compared to that after exposure to EVs obtained at time point I, in both subgroups of patients: neo-adjuvant, *p*=0.0005; adjuvant *p*=0.05) (Figure 6d).

## DISCUSSION

The current study is the first to explore the impact of the primary tumor and various types of chemotherapy on circulating EVs. This was achieved by characterizing EVs of BC patients, both in patients with primary tumors before (I) and at the end of (II) chemotherapy (neo-adjuvant chemotherapy), and in patients who underwent surgical removal of their primary tumor before (I) and at the end of (II) chemotherapy (adjuvant chemotherapy).

Indeed, these are not the only factors that may affects EVs characteristics; different types/stages of breast cancer can strongly determine the circulating vesicles and thus act as confounding factors, and in some cases we were able to emphasis the effects of disease severity.

EVs, exhibiting endocrine and paracrine effects, serve as a transporter of molecular cargo between cells [17]. The larger size of patient EVs, found mainly in the neo-adjuvant patients following chemotherapy, is associated with their increased transport capacity, emphasizing the impact of the existing tumor and exposure to chemotherapy on EV characteristics.

### Tumor markers

A trend of increase was found in EVs expressing of the tumor marker EpCAM in the neo-adjuvant patients before chemotherapy, which may indicate tumor existence in this patient group. Nevertheless, only 38% of neo-adjuvant patients presented higher levels of EVs-EpCAM expression than the highest value found in healthy controls, which is probably not sufficient to use for early diagnosis of all cases of BC. A further increase in EpCAM was found in the neo-adjuvant patients at the end of chemotherapy, significantly higher than in HC, probably related to the apoptotic effect of the chemotherapy on tumor cells, not existing in the adjuvant group. However, a trend of increase was found also in the EpCAM-bearing EVs before chemotherapy in the adjuvant group. We can assume that tumor cell EVs remained in the circulation of the adjuvant patients from the pre-surgery period. Their natural clearance from the circulation over time resulted in their decrease at the end of chemotherapy. EpCAM is expressed on epithelial cells, is highly up-regulated in epithelial carcinomas, and is associated with cancer progression and metastases. It is a transmembrane glycoprotein, involved in cell-cell interactions, migration, proliferation, differentiation and cellular signaling [18]. Tumor-derived EVs have recently been shown to contribute to tumor re-growth, partially by inducing mobilization and tumor homing of specific bone marrow derived pro-angiogenic cells [19]. Therefore, monitoring of tumor EVs, and especially EPCAM, may have important value.

In the current study, no significant defenses were found in the expression of MUC1 on the study cohort EVs. Mucins1 (MUC1) is present in healthy epithelial cells and over-expressed in ∼90 % of breast carcinomas, correlating with poor prognosis and an increased risk of metastasis. In BC, MUC1 becomes hypo-glycosylated compared to normal epithelial cells, resulting in less branching and a shorter length of the oligosaccharide chains. These shortened oligosaccharides can bind endothelial E-selectin, enabling BC cells to penetrate the EC in the vessel wall via a similar mechanism used by leukocytes [20] and inducing EC injury. In the current study, there is a high level of EV-bearing MUC1 in the adjuvant patients that may be related to the injured epithelial cells from the surgery while, after chemotherapy, MUC1-EVs population probably originated from normal epithelial cells. Therefore, it can be concluded that MUC1-EVs cannot serve as a biomarker for existing tumors.

### Endothelial EVs

We found high levels of endothelial markers, such as VE-cadherin (vascular endothelial cadherin, CD144), E-selectin, (CD62E), and the endothelial protein c receptor (EPCR), that may indicate vascular damage even before exposure to chemotherapy, as demonstrated in other pathologies [21, 22]. Vascular damage and increase in endothelial EVs also related to increase of age as we previously demonstrated [23]. That may explain the wide range and the variability in the control group which partially overlap with patients results.

Previous studies found that interaction of BC cells with endothelial cells leads to phosphorylation of VE-cadherin and disruption of endothelial adherent junction [24]. High levels of serum VE-cadherin were significantly correlated to a shorter progression-free and overall survival [25]. We can assume that the presence of tumor and tumor-related EVs in the neo-adjuvant patients or a short period after surgery in the adjuvant patients induced tissue and vascular damage prior to chemotherapy. The impact of chemotherapy on vascular damage reflected in the high levels of endothelial EVs, which was documented without tumor existence and a few month after surgery were wounds probably healed, in the adjuvant patients at samples of time point II.

### EVs thrombogenicity

The current study is the first to describe EVs thrombogenicity with chemotherapy in adjuvant and neo-adjuvant BC patients. EVs obtained before chemotherapy from the neo-adjuvant patients and from adjuvant patients were significantly more thrombogenic than EVs obtained from HC. They expressed higher levels of negatively charged phospholipids, a significant increase in TF, a decrease in TFPI resulting in an increase of the TF/TFPI ratio, and a significant increase in EPCR. The latter is related not only to vascular endothelia damage but also to an increase in thrombogenicity, probably as the soluble EPCR serves as a procoagulant factor [26]. Exposure to chemotherapy further enhanced patient EVs procoagulant activity, probably as a result of reduction in the anticoagulant TFPI that also indicates vascular cells damage. The high inverse correlation that was found between disease stage in neo-adjuvant patients at the end of chemotherapy further emphasizes and strengthens the impact of the combination of tumor and chemotherapy on EVs thrombogenicity. We also found that chemotherapy induced accumulated effects on EVs thrombogenicity during the long treatment period, and that the short-term effect is dominant as well, and dependent on the type of chemotherapy agent. The first 24hours after treatment with Adriamycin and cyclophosphamide demonstrated a powerful effect on EVs thrombogenicity and induced a major increase in the EVs TF/TFPI ratio. In contrast, 24hours of exposure to paclitaxel had a minimal effect on EVs thrombogenicity. The special results obtained just 24hours post administration of Adriamycin suggest that certain changes in EVs following chemotherapy are time dependent. More specifically, more immediate and shorter term effects than those discussed in the current study may occur following their measurement toward the end of chemotherapy treatment. These effects could be non-cumulative, thus missed from observation by conducting just a single and late measurement. This possibility would imply that future studies on EVs and their correlations with chemotherapy should include also measurements closer to the time of chemotherapy administration.

In the current study, 4/54 (7%) of patients developed DVT, a similar rate to that reported in previous studies [27]. Additional increases were found in TF/TFPI ratio at the end of chemotherapy in 65% of neo-adjuvant patients and 40% of adjuvant patients, presenting a significant increase in EVs pro-coagulant activity at the end of chemotherapy. The ability to predict the risk for thrombotic events is missing. A recent large study on BC patients recognized a risk of VTE equivalent to 6% yearly while undergoing chemotherapy and in the month after treatment [5, 28]. EV TF/TFPI ratio and EVs pro-coagulant activity may predict a tendency to a hypercoagulable state and may be used as a marker or “red flag” in specific cases, indicating a higher risk for a thrombotic event.

Exposure of endothelial cells (*in vitro*) to patient EVs increased cells pro-coagulant activity. The most significant effect was related to the EVs obtained from the adjuvant patients at the end of chemotherapy, which may relate not only to the increase in EVs thrombogenicity but also to the content of pro-inflammatory proteins that mediate crosstalk among thrombosis, inflammation, and vascular dysfunction-characterized cancer patients [29, 30].

### EVs and angiogenesis

In the current study, the expression of both VEGF receptors (VEGF-R), KDR and FLT1, on EVs was found to be similarly high in all patient sub-groups compared to HC and was not affected by tumor existence or exposure to chemotherapy. It was documented that expression of VEGF-R on patient EVs correlated with the increase of soluble FLT-1 in the serum of BC patients but not in HC [31]. VEGF-R regulates the formation of blood and lymphatic vessels and is expressed on endothelial cells as well as BC cell lines. KDR directly regulates tumor angiogenesis and its high expression correlated with BC lymph node metastasis [32]. We can assume that the high levels of VEGF-R bearing EVs in the circulation reflects their expression on the tumor and on damaged vascular cells, and gives them the opportunity to reach a new niche, transferring those receptors to recipient cells that may support angiogenesis and metastasis.

We found that EVs obtained from the neo-adjuvant patients before chemotherapy contained higher levels of growth factors compared to adjuvant patient EVs. We can assume that the relative high content in growth factors in the neo-adjuvant EVs is associated with the presence of tumor cells. The growth factors that were high in the EVs of neo-adjuvant patients (Angiogenin, EGF, PDGF-BB, PECAM-1, GRO, MMP-9) are known to be involved in angiogenesis, tumorigenesis and metastatic progression [33–36], and probably have pathophysiological influences. The pro-inflammatory proteins (RANTES CXCL5, IL6) may result from the “host” response tumor cells, mainly injured endothelial cells, as reflected by the high prevalence of endothelial EVs. In the adjuvant EVs, we found increased pro-inflammatory proteins and growth factors at the end of chemotherapy. Response to chemotherapy contributes not only to tumor cells but also to the tumor-environment and host cells response [37]. For example, exposure of non–tumor-bearing mice to paclitaxel induced IL-1b production [38]. The current study demonstrated changes in the content of growth and inflammatory factors in EVs obtained from adjuvant patients from different patient groups after chemotherapy, which emphasize this phenomenon.

### EVs effects on endothelial function

Healthy endothelium characterized by anticoagulant and anti-inflammatory nature, were endothelial dysfunction reduced vasodilation and shifting cells to pro-inflammatory and pro-thrombic states [39]. Cell origin and the stimulation used for their generation will determine the EVs balance between proangiogenic proteins and inflammatory proteins and their effects on endothelial cells [40]. By exposure of EC (*in vitro*) to patients EVs obtained from different sub groups and disease states, we emphasize the effects of the high content of growth factors in EVs obtained from neo-adjuvant patients, probably affected by the existing tumor. These EVs secured endothelial cells from apoptosis but also reduced cell proliferation and induced massive cell migration. This is not the first time for such contrary results; previously we reported that inflammatory monocyte EVs (resulting from exposure to lipopolysaccharides) disrupt EC integrity leading to two contradicting outcomes – tube formation and apoptosis [23]. An earlier study found that blood vessels can split into new vessels without endothelial cells proliferation, a process that has been demonstrated in various tumors [41]. The prevention of apoptosis by neo-adjuvant EVs probably enable their intensive migration which related to angiogenesis.

In contrast to neo-adjuvant EVs, the adjuvant EVs contained more inflammatory proteins affected from the recent surgery and the wound healing process which regulated, by high content growth factors, cytokines and chemokines [42], some of which were packed and transferred via EVs [43]. These adjuvant EVs induce more apoptosis and less proliferation and migration compared to HC-EVs.

The characterization of EVs obtained from BC patients while receiving chemotherapy in both subgroups of patients, both before and after removal of all apparent disease, allowed a new insight on endothelial damage related to the long-lasting influences of the tumor. EVs cytokine/growth factors content and their angiogenic effects on EC make them an important player in the “rebound vasculature” after chemotherapy [19, 44]. EVs thrombogenicity related to tumor or surgery is further enhanced by chemotherapy and the pro-inflammatory process that succeeds breast surgery. These findings support further elucidation of EVs properties for early recognition of pro-thrombotic markers that may help to identify patients at higher risk and improve their care accordingly.

Despite the fact that the results often show an overlapping between controls and patients, there were some patients who did not have abnormal EVs profile and some patients who have results which are significantly higher than the highest value found in controls as a result of an existing tumor or as secondary effects of the injured tissues as a result of surgery. We found that about 50% of patients presented significantly higher levels of endothelial markers and indicate higher thrombogenic states which further intensify after chemotherapy, pointing to the impact of the treatment on EVs features. However, we must remember that there are probably additional individual parameters related to each person's genetic background, inflammatory condition, and other factors at that time of sampling that can affect EVs profile. All this requires additional studies.

The new tumor staging in breast cancer remains based on tumor, node, metastasis (TNM) anatomic factors. However, recognition of the prognostic influence of grade, hormone receptor expression, HER2 amplification, tumor biomarkers and low Oncotype DX recurrence scores mandated their inclusion in the staging system [45]. Recently, more complicated systems, such as a novel gene panel of cell-free DNA methylation markers strongly predict survival outcome in metastatic breast cancer and may have clinical usefulness in risk stratification and disease monitoring [46].

The identification of abnormal extracellular vesicles profiles may also help by pointing to some peculiar neoplastic pathophysiology which should benefit from additional specific treatments. However, the hypothesis that it could be a tool allowing moving towards patient-based tailored treatment is still highly speculative. In the current study, the patients had a very good prognosis as a group that does not allow conclusions in this direction.

In summary, breast cancer is approached as a systemic disease, bearing undetectable micro-metastases at the time of diagnosis and associated with a hypercoagulability state which is affected by chemotherapy [5]. Circulating EVs can reflect and affect disease dynamic and thrombogenicity and, therefore, may be used as a biomarker for hypercoagulability states [11].

## MATERIALS AND METHODS

### Study design and setting

This study was conducted between 2009 and 2015. It was approved by the institutional review boards of Rambam Health Care Campus (Approval No. 0368-09-RMB) and Ha'emek Medical Center (Israel Ministry of Health Approval No. 920090920). The BC population (n=54) was composed of two subgroups of patients: 1) Patients after biopsy only (n=25), receiving chemotherapy as neo-adjuvant treatment, and 2) Patients after surgical removal of the tumor (n=29), receiving chemotherapy as adjuvant treatment. Table 1 describes patient characteristics and chemotherapy variations.

Blood samples were obtained from each patient both before the first chemotherapy cycle (time point I) and on the day of last cycle (time point II). Samples were also collected from some of the study population at 24 hours post time point I, following the first treatment with Adriamycin and cyclophosphamide, and 24 hours following the first treatment with paclitaxel. In addition, one blood sample was collected from each one of the age-matched healthy controls.

### EVs isolation

Fifteen ml of peripheral venous blood were drawn from study participants into sodium citrate (3.2%) tubes; platelet-poor plasma (PPP) was obtained after two centrifugations (15 minutes, 1500g) within an hour of collection and frozen at −80°C. EVs were isolated from thawed PPP by centrifugation (1 hour, 20000g). Supernatant liquid was discarded, and EV pellets were used for cell culture stimulation. Only part of the obtained samples was used in every assay.

### EVs characterization

The size of circulating EVs obtained from the study population were measured using Nanoparticle Tracking Analysis (NTA, Version 3.1 Build 3.1.54) (Nanosight NS500, Amesbury UK). Each sample was studied three times/60-second video using sCMOS camera at level 16 (Slider Shutter: 1300), each time at a different position in the chamber. All measurements were performed at 25°C according to the published protocol [47].

Each video was analyzed by the NTA software (NTA 3.1, NanoSight Ltd.); Detect threshold: 5, Blur Size: Auto, Max Jump Distance: Auto: 10.9-18.3 pix.

Antigen levels of EVs were evaluated by flow cytometry (FACS-CyAn ADP analyzer, Beckman Coulter). EVs were labeled with Fluorescein isothiocyanate (FITC)-Annexin V (Bender MedSystems, Austria) that binds to negatively charged phospholipids and to specific fluorescent antibodies: FITC-CD235 (red blood cells marker), Allophycocyanin (APC)-Flt-1 (vascular endothelial growth factor receptor (VEGFR)-1), PE-KDR (VEGFR-2), APC IgG1κ Isotype control (R&D Systems, Minneapolis MN), Phycoerythrin (PE)-CD41 (platelet marker), PE-Cd62P (activated platelet marker), PE-anti CD62E (E-selectin), APC-anti CD144, PE-anti-human CD227 (Mucin-1), and anti-Epithelial Cell Adhesion Molecule (EpCAM. bio-Legend, CA). Coagulation markers: FITC anti-human TF and anti-human TFPI (America Diagnostica, CA). Anti-mouse IgG-PE (Jackson PA), IgG isotype controls FITC, and PE were purchased from BD Pharmingen, CA.

Pro-coagulant activity was evaluated using the Factor X active (FXa) chromogenic assay [23]. The results were converted to TF arbitrary units (AU).

EVs proteins content were screened by the Human Angiogenesis Protein Antibody Array (Ray Bio, Georgia, USA). EV protein extract was obtained from a pool of four specimens within each patient sub-group and quantified using the bicinchoninic acid (BCA) protein quantification kit (Thermo Fisher Scientific Inc, Illinois, USA). Each protein array slide was loaded with 25μg of protein from the EVs pool lysate, and the array was performed according to the manufacturer's instructions. Expression of each protein was represented in duplicate on the array slides. Duplicate dots identifying each protein were scanned and quantified by Total lab software. The mean fluorescence intensity of these dots (AU) was determined for intergroup comparisons.

### Endothelial cell culture

Human umbilical vein endothelial cells (HUVECs) were isolated from umbilical cords obtained at term of normal pregnancy, according to the previously described technique [23]. Passages 4 to 8 were used to assess the effects of patient EVs on endothelial cells (EC) thrombogenicity, apoptosis versus proliferation and migration.

### Effects of circulating EVs on endothelial cells culture – *in vitro*

### EC apoptosis

HUVEC and and EVs pellet (isolated from 2 mL of PPP obtained from several individual samples) were co-seeded in 24-well tissue culture plates for 20 hours. Cells treated with 50U of DNAase (Sigma-Aldrich, Israel) for 10 minutes served as a positive control. Then, cells were washed, and the TUNEL (terminal deoxynucleotidyl transferase dUTP nick end labeling) assay (Roche Diagnostics, Mannheim, Germany) was performed according to the manufacturer's instructions. Acquisition was performed using flow cytometry analysis device. Results were expressed as percentage of TUNEL positive cells out of the total cell population in each well.

### EC proliferation

HUVEC were seeded in 96-well tissue culture (5000 cells/well) in 100μl of growth medium. After 24 hours, the medium was replaced by EV pellets isolated from 0.5mL of PPP obtained from several individual samples and compared to untreated cells. After 20 hours, 50μl of the reaction solution of XTT [2,3-bis(2-methoxy4-nitro-5-sulfophenyl)-2H-tetrazolium-5-carboxanilide, Biological Industries, Israel)] were added to each well for 0.5 hour. The absorbance of the samples was measured using the ELISA reader (450/630 nm).

### EC migration

Cell migration was measured using 24-transwell inserts (BD Biosciences). HUVEC were seeded on the upper chamber membrane, while EVs were added to the lower chamber (medium without EVs and used as controls). After 24 hours, the inserts were fixed with 4% formaldehyde and stained with 0.5% crystal violet for 10 minutes each. Cells on the top of the membrane were removed, and the remaining cells on the bottom side of the membrane were photographed using inverted microscopy.

### Statistical analysis

The differences between patient groups parameters (stage, grade and tumor size) were analyzed by Fisher exact test for a 2 × 2 contingency table. EVs characteristics were analysed by GraphPad 5 software. Results were assessed by 1-way ANOVA, Bonferroni multiple comparisons test. When only two groups were compared, t test was used. P<0.05 was considered statistically significant. The results were expressed as a means ± SD.

## Abbreviations

APC: allophycocyanin
AU: arbitrary units
BC: breast cancer
BCA: bicinchoninic acid
DVT: deep vein thrombosis
EC: endothelial cells
EGF: epidermal growth factor
EpCAM: epithelial cell adhesion molecule
EPCR: endothelial protein C receptor
EVs: extracellular vesicles
FACS: flow cytometry
FITC: fluorescein isothiocyanate
FXa: Factor X active
GRO: growth-related oncogene
HER2: human epidermal growth factor receptor 2
HUVEC: human umbilical vein endothelial cells
IL6: interleukin 6
MMP-9: matrix metallopeptidase 9
MPs: microparticles
Muc1: Mucin-1
NTA: Nanoparticle Tracking Analysis
PDGF: platelet drive growth factor
PE: phycoerythrin
PECAM-1: platelet endothelial cell adhesion molecule
PPP: platelet-poor plasma
RANTES: Regulated on Activation, Normal T Cell Expressed and Secreted
TF: tissue factor
TFPI: tissue factor pathway inhibitor
TNM: tumor, node, metastasis
TUNEL: terminal deoxynucleotidyl transferase dUTP nick end labeling
VEGFR: vascular endothelial growth factor receptor
VTE: venous thromboembolism.
